# Acoustic Analysis of Voice in School Teachers

**Published:** 2018-06-30

**Authors:** Nain Bahadur Mahato, Deepak Regmi, Meera Bista, Pema Sherpa

**Affiliations:** 1 Department of ENT-HNS, Kathmandu Medical College, Sinamangal, Kathmandu, Nepal

**Keywords:** *acoustic analysis*, *doctor speech*, *fiberoptic laryngoscopy*, *GRBAS scale*, *voice*

## Abstract

**Introduction:**

The term ‘voice’ is the acoustic energy generated from the vocal tract that are characterized by their dependence on vocal fold vibratory pattern. Teachers as professional voice users are afflicted with dysphonia and are discouraged with their jobs and seek alternative employment. Loud speaking and voice straining may lead to vocal fatigue and vocal fold tissue damage. The objective of the study is to assess the quality of voice of school teachers before and after teaching practice.

**Methods:**

Sixty teachers from various schools, volunteered to participate in this study. Acoustic analysis Doctor Speech Tiger Electronics, USA was used to assess the voice quality of the school teachers before and after teaching practice. The data were collected and analyzed using Doctor Speech Tiger Electronics, USA. Analysis was performed in terms of perturbation (jitter and shimmer), fundamental frequency, harmonic to noise ratio and maximum phonation time.

**Results:**

We found statistically significant difference in all the four parameters except the Jitter value. The fundamental frequency and shimmer value has significantly increased (P<0.001) and (P=0.002) respectively after teaching practice. Unlikely, there was significant decrease in harmonic to noise ratio value (P<0.001) and maximum phonation time value (P<0.01) after teaching practice.

**Conclusions:**

Vocal abuse, overuse, or misuse in teaching practice over a long period of time can result in inadequate phonatory pattern due to vocal fold tissue damage, which ultimately results in vocal nodules or polyps. So voice evaluation is particularly important for professional voice users and for the people who are concerned about their quality of voice.

## INTRODUCTION

The term ‘voice’ is the acoustic outputs from the vocal tract that are characterized by their dependence on vocal fold vibratory inputs'.^1^ Professional voice users means those who have consistent and appealing voice quality as a primary tool.^2^

Many questionnaire studies have reported, 50-80% of the teachers have voice problems,^3,4^ and teaching profession is one of the 10 occupations that require medical help for voice problems,^5,6^ which can reduce their professional effectiveness.^7–8^ Voice disorders are twice in female compared to the male teachers,^9,10^ and excessive voice abuse causes hoarseness, vocal fatigue or even aphonia.^11^ Teachers are more at risk of developing voice disorders than others,^6,12,13^ and the prevalence is significantly higher among teachers (57.7%) than in non-teachers (28.8%). Non-treated functional dysphonia causes irreversible laryngeal lesions leading to hoarseness.^14^

The objective of our study is to assess the quality of voice in school teachers before and after teaching practice.

## METHODS

This Descriptive Cross-Sectional Study was done in the department of ENT-HNS of KMCTH from 3^rd^ Jan 2018 to 25^th^ March 2018 AD. The approval for ethical clearance was taken from ethical committee for research and development council of Kathmandu Medical College Teaching Hospital. Written consents were taken from the participants. Sample size was calculated using the formula:

$$\begin{array}{l} {\text{Sample size = Z}}^{2}{\text{×PQ/d}}^{2}\\ \text{Confidence Interval (CI) = 95\%}\\ \text{Error (d) = 10\%}\\ \text{Prevalence (P) = 16}.33\\ {\text{So, Sample size = Z}}^{2}{\text{×PQ/d}}^{2}\\ \text{= 1 .96 × 1 .96 × 0 .1633 × (1-0 .1633)/(0}{\text{.1)}}^{2}\\ \text{= 0 .62733 × 0 .8367/0}.01\\ \begin{array}{l} \text{= 52}.4887\\ \text{Therefore, the calculated sample size was 53 .} \end{array} \end{array}$$

Sixty teachers from various schools, age ranging from 22-40 years with 3-10 years of teaching experience volunteered to participate in this study. All the teachers to be enrolled in the study group were examined by the consultant otolaryngologists. All the teachers had undergone subjective analysis (GRBAS scale) and fiberoptic laryngoscopic (FOL) examination to assess the vocal cord mobility and vocal cord pathology before undergoing acoustic analysis. Out of 60 teachers, 7 (11.6%) teachers were excluded from the study because of common cold and 4 (6.6%) of them had tiny vocal nodules for which they underwent speech therapy as treatment protocol. Acoustic analysis {Doctor Speech (DRS) Tiger Electronics USA} was used to assess the voice quality of the school teachers before and after teaching practice. The data were collected and analyzed using Doctor Speech (DRS) Tiger Electronics, USA. All the data were recorded in a sound proof room. Voice recording was done using microphone which was set at a distance of approximately 8 cm from the upper lip, and the person sitting in comfortable position, so that any distortions or modifications in the recording could be avoided. After 3 training emissions, the teacher was asked to sustain the vowel /i/ as long as steadily possible. All the voice recordings were repeated for three times and to avoid voice onset effects, first 500ms of the voice data were not included. Recordings were begun after initiation of voicing and ended before the patient terminated voicing. An interval of 3 seconds from the mid portion of each sample was selected for acoustic analysis. Analysis was performed in terms of perturbation (jitter and shimmer), fundamental frequency (F_0_), harmonic to noise ratio (HNR) and maximum phonation time (MPT). Statistical analysis was performed using SPSS version 18 statistical software and the level of significance was set at 0.05.

## RESULTS

We found statistically significant difference in all the four parameters except the Jitter value. The fundamental frequency has significantly increased (P<0.001) after teaching practice 263.016 (52.095) than before teaching practice 242.112 (51.021). Similarly, Shimmer value has also significantly increased (P = 0.002) after teaching practice 1.132 (0.660) than before teaching practice 0.930 (0.451). There was significant decrease (P<0.001) in HNR value after teaching practice 29.810 (3.753) than before teaching practice 32.423 (2.956). Likewise, significantly decrease (P<0.01) in MPT value after teaching practice 15.501 (2.405) than before teaching practice 23.125 (1.201) was found in our study (Table 1).

**Table 1.** Difference in acoustic parameters before and after teaching practice.

Parameters	Before teaching practice, Mean (SD)	After teaching practice, Mean (SD)	P (*denotes significant P)
Jitter%	0.195 (0.186)	0.215 (0.198)	0.22
Shimmer%	0.930 (0.451)	1.132 (0.660)	0.002*
HNR	32.423 (2.956)	29.810 (3.753)	<0.001*
F (Hz) o	242.112 (51.021)	263.016 (52.095)	<0.001*
MPT (s)	23.125 (1.201)	15.501 (2.405)	<0.01*

Out of the sixty teachers, evaluated by two of the surgeons and one speech pathologist, 7 (11.6%) of them had abnormality in voice parameters in pre-assessment period; while 4 (6.6%) of them had tiny vocal nodule confirmed by FOL, for which they underwent speech therapy as treatment protocol. In pre-assessment period, 7 (11.6%) of the teachers had abnormality of Grade and Roughness, 4 (6.6%) had Breathiness, 3 (5%) had Asthenia and 4 (6.6%) had Strain. After three weeks of speech therapy, only 1 (1.6%) teacher had persistent mild abnormality (Grade, Roughness) at 3 weeks follow-up (Table 2).

**Table 2.** Showing number of school teachers with voice disorders in GRBAS scale.

Parameters (GRBAS)	Pre-speech therapy (No. of teachers)	3 weeks post speech therapy (No. of teachers)
Grade	7	1
Roughness	7	1
Breathiness	4	0
Asthenia	3	0
Strain	4	0

## DISCUSSION

As we know that most of the school teachers often speak loudly for a longer duration in presence of high background noise because of this, most teachers suffer from vocal fatigue at the end of the workday. The objective evaluation of voice via acoustic analysis seems to be of particular value because it is non-invasive and relatively easy to perform. ^15,16^ Acoustic analysis is a useful tool for assessing the voice quality and evaluating the effectiveness in voice therapy.^17^ In our study, we found that there were significantly different values on all acoustic parameters of voice except the jitter. In a field study conducted by Rajasudhakar and Savithri in five elementary school teachers, reported that after 6 hours of teaching, fundamental frequency of phonation, jitter, and speaking fundamental frequency were increased compared to pre-teaching condition.

In our study, the measure of F_0_ in teachers was higher after teaching practice at the end of workday than before teaching {before teaching F_0_ = 242.1 12 (51.021), after teaching F_0_ = 263.016 (52.095); P< 0.001}. Our study showed similar results as that of the study done by Stempleet al^18^ and Vilkman et al.^19^ The increment in F_0_ value according to Stemple et al^18^ is due to weakness of the thyroarytenoid muscle. When the muscular layer of the thyroarytenoid muscle slackens, the cover and transition layers of the vocal folds stiffen which leads to increased rate of vibrations and a rise in F_0_. Similarly, there was significant increase in shimmer value after teaching practice than before teaching {before teaching 0.930 (0.451), after teaching 1.132 (0.660); P = 0.002}, however, no significant increase in jitter value was seen. This increment in perturbation measures were similar to the study done by Samuel et al.^20^ Increased Jitter or shimmer values have been associated with phonatory instability due to ageing and various laryngeal pathologies. ^21^ This change in jitter or shimmer values is due to lowered muscle tonus and impaired neuromotor control of the larynx because of fatigueness of vocal cords.

The HNR value was significantly reduced at the end of the workday {before teaching 32.423 (2.956), after teaching 29.810 (3.753); P<0.001} which is similar to the study done by Vertraete et al, ^22^ who reported decrease of HNR in female teachers after a day work. Inadequate glottic closure or aperiodic vocal fold vibration, allows excessive airflow through the glottis, giving rise to turbulence and hence resulting in a higher noise level in the spectrum, thus reflecting in a lower HNR.^23^ In one study, it was found that more professional teachers had lower level of HNR value.^24^ We found significant reduction of maximum phonation time (MPT) at the end of workday {before teaching 23.125 (1.201), after teaching 15.501 (2.405); P<0.01}. This decreased MPT values are suggestive of transglottic leakage of air during phonation and increased values are suggestive of increased glottal closure and increased muscle tension. ^25,26^

## CONCLUSIONS

Teaching profession is one of the most vulnerable occupations that require medical help for voice problems. Loud speaking and voice straining in teaching practice over a long period of time can result in inadequate phonatory pattern with excessive musculoskeletal tension, which ultimately results in vocal fatigue and vocal fold tissue damage. Acoustic analysis is a useful non-invasive tool for assessing the voice quality and evaluating the effectiveness in voice therapy. So, the objective evaluation of voice via acoustic analysis is particularly important for professional voice users and for the people who are concerned about their quality of voice for the early diagnosis and proper treatment.
